# A Single Divergent Exon Inhibits Ankyrin-B Association with the Plasma Membrane

**DOI:** 10.1074/jbc.M113.465328

**Published:** 2013-04-08

**Authors:** Meng He, Wei-Chou Tseng, Vann Bennett

**Affiliations:** From the ‡ Department of Pharmacology and Cancer Biology,; § Howard Hughes Medical Institute, and; ¶ Departments of Biochemistry, Cell Biology, and Neurobiology, Duke University, Medical Center, Durham, North Carolina 27710

**Keywords:** Epithelial Cell, Evolution, Neurons, Plasma Membrane, Transfection, Ankyrin Diversity, Autoinhibition, Intramolecular Interaction

## Abstract

Vertebrate ankyrin-B and ankyrin-G exhibit divergent subcellular localization and function despite their high sequence and structural similarity and common origin from a single ancestral gene at the onset of chordate evolution. Previous studies of ankyrin family diversity have focused on the C-terminal regulatory domain. Here, we identify an ankyrin-B-specific linker peptide connecting the ankyrin repeat domain to the ZU5_2_-UPA module that inhibits binding of ankyrin-B to membrane protein partners E-cadherin and neurofascin 186 and prevents association of ankyrin-B with epithelial lateral membranes as well as neuronal plasma membranes. The residues of the ankyrin-B linker required for autoinhibition are encoded by a small exon that is highly divergent between ankyrin family members but conserved in the ankyrin-B lineage. We show that the ankyrin-B linker suppresses activity of the ANK repeat domain through an intramolecular interaction, likely with a groove on the surface of the ANK repeat solenoid, thereby regulating the affinities between ankyrin-B and its binding partners. These results provide a simple evolutionary explanation for how ankyrin-B and ankyrin-G have acquired striking differences in their plasma membrane association while maintaining overall high levels of sequence similarity.

## Teaser

**Background:** Ankyrin-B is intracellular, whereas ankyrin-G associates with plasma membranes.

**Results:** An ankyrin-B-specific linker between the ANK repeat domain and the ZU5_2_-UPA module inhibits ankyrin-B binding to membrane protein partners and localization to plasma membranes of epithelial cells and neurons.

**Conclusion:** The linker region contributes to functional differences between ankyrins.

**Significance:** This work explains the distinct behavior of ankyrins regarding plasma membrane localization.

## Introduction

The vertebrate ankyrin family contains three members, ankyrin-R, ankyrin-B, and ankyrin-G, that are encoded by distinct genes (ANK 1, 2, and 3) with distinct cellular location and functions (1). Vertebrate ankyrins evolved from a single ankyrin gene in the invertebrate urochordate *Ciona intestinalis* and, overall, are highly conserved in primary sequence and domain organization (2). The N-terminal ANK repeat domain (membrane-binding domain) is involved in diverse protein-protein interactions (3) and also is *S*-palmitoylated at a conserved cysteine (4). Following the ANK repeat domain, there is a ZU5_2_-UPA domain module, which includes the β-spectrin binding site (5–7). A death domain of unknown function is also present in all three ankyrin members. A C-terminal regulatory domain exhibits the highest diversity among the three ankyrins (1, 8, 9). The ankyrin-B C-terminal region interacts with its ANK repeat domain and plays a critical role in regulating ankyrin-B functions in cardiomyocytes (10, 11).

Ankyrin-B and ankyrin-G are coexpressed in most tissues but have distinct cellular localization and function. In rod photoreceptors, ankyrin-G exclusively localizes at the outer segment and is required for segregating cyclic nucleotide-gated channels to the outer segment (12), whereas ankyrin-B is expressed in the inner segment, where it is required for localization of the sodium/calcium exchanger and Na/K ATPase (13). In skeletal muscle, ankyrin-B is required for delivery of β-dystroglycan to costameres and for a costamere-associated population of microtubules, and ankyrin-G is required to retain β-dystroglycan and dystrophin at costameres (14). Additionally, ankyrin-G is a master organizer of the axon initial segment and nodes of Ranvier (15–18), and ankyrin-B localizes to the distal axon together with αII-spectrin and βII-spectrin (19).

The molecular mechanisms underlying distinct functions of ankyrin-B and ankyrin-G remain to be fully elucidated. A previous study showed that the C-terminal region determines the specificity of ankyrin-B function in cardiomyocytes (11). The ZU5_2_-UPA module determines the distal axonal localization of ankyrin-B in neurons (19). Here, we adopted a comprehensive domain substitution strategy to determine effects of exchanging domains or unstructured regions between ankyrin-B and ankyrin-G on their ability to associate with epithelial lateral membranes. This systematic approach led to identification of a heretofore unrecognized ankyrin-B-specific linker peptide connecting the ANK repeats and ZU5_2_-UPA module that prevents association of ankyrin-B with plasma membranes of epithelial cells and neurons through intramolecular association with ANK repeats.

## EXPERIMENTAL PROCEDURES

### 

#### 

##### Reagents

Dynabeads® protein G for immunoprecipitation (catalog no. 10004D) and Lipofectamine® 2000 transfection reagent (catalog no. 11668-027) were purchased from Invitrogen. QuikChange II XL site-directed mutagenesis kits (catalog no. 200522) were purchased from Agilent Technologies. Mouse monoclonal anti-HA antibody (catalog no. H6908) was purchased from Sigma-Aldrich. Rabbit polyclonal anti-GFP antibody was laboratory-generated. Rabbit polyclonal anti-ankyrin-B or anti-ankyrin-G antibody was generated using the C-terminal domain as antigen.

##### Cloning of Chimeric Ankyrin-B/G Constructs

The first generation of chimeric GFP-tagged ankyrin-B/G constructs was described previously (11). In ankyrin-B, the linker region was defined as amino acids 848–960, the ZU5 domains were defined as amino acids 961–1287, and the UPA domain was defined as amino acids 1288–1443. In ankyrin-G, the linker region was defined as amino acids 874–984, the ZU5 domains were defined as amino acids 985–1312, and the UPA domain was defined as amino acids 1313–1476. Two restriction sites, SphI and SpeI, were introduced after the linker region and the second ZU5 domain, respectively. The corresponding regions in ankyrin-G and ankyrin-B were exchanged using standard cloning protocols.

##### Transfection and Immunostaining

10^5^ Madin-Darby canine kidney cells were plated on MatTek plates, and the next day the cells were transfected with 50 ng of plasmids using Lipofectamine® 2000 following the recommended protocol. 24 h after transfection, cells were washed with cold PBS, fixed with 4% paraformaldehyde at room temperature for 15 min, and permeabilized with 0.2% Triton X-100 at room temperature for 10 min followed by a 30-min blocking in PBS buffer containing 4% bovine serum albumin. Then cells were incubated with primary antibody at 4 °C overnight. The next day, cells were washed with PBS buffer three times and then incubated with fluorescence-conjugated secondary antibodies (Alexa Fluor 488, 568, or 663) at room temperature for 2 h. Fluorescent antibody labeling was visualized using Zeiss LSM 510 or 780 laser scanning microscopes. Images were collected using a ×63 numerical aperture 1.4 objective lens, and XZ planes were reconstructed from Z stacks with optical sections of 0.5 μm.

##### Membrane Recruitment Assay

This assay was reported previously (20). Briefly, 10^5^ HEK293 cells were plated in collagen-coated MatTek plates. The next day, cells were cotransfected with 100 ng HA-tagged neurofascin-186 or E-cadherin plasmids and 80 ng GFP-tagged chimeric ankyrin plasmids. 24 h later, cells were fixed and processed for immunofluorescence as described above.

##### Coimmunoprecipitation

6 × 10^6^ 293T cells were plated in 10-cm dishes, and the next day cells were cotransfected with 4 μg of plasmid DNA encoding the HA-tagged ANK repeat domain of ankyrin-B or ankyrin-G with 2 μg of plasmid DNA encoding GFP-tagged B-linker. 48 h after transfection, cells were collected and lysed in lysis buffer (10 mm sodium phosphate, 2 mm NaEDTA, 0.32 m sucrose, and protease inhibitors (10 μg/ml AEBSF, 30 μg/ml benzamidine, 10 μg/ml pepstatin, and 10 μg/ml leupeptin) (pH 7.4)) by passage through a 27-gauge needle. Cell lysates were then centrifuged at 10^5^ × *g* for 30 min, and the supernatant was collected and incubated with protein G dynabeads preloaded with anti-GFP antibody. Immunoprecipitation samples were then analyzed by SDS-PAGE and Western blotting.

##### Hippocampal Neuronal Cultures and Transfection

Neurons and medium were prepared as described (21). Briefly, hippocampi of postnatal day 0 mouse pups were isolated, treated with trypsin, and then gently triturated through a glass pipette with a fire-polished tip. The dissociated cells were plated onto poly-d-lysine- and laminin-coated MatTek dishes in Neurobasal-A medium containing 10% fetal bovine serum, B27 supplement, 2 mm glutamine, and penicillin/streptomycin. On the following day, the medium was replaced with fresh serum-free Neurobasal-A medium containing B27, glutamine, and Ara-C. Cultured hippocampal neurons at day 5 were transfected with 50 ng of chimeric ankyrin plasmids following the standard protocol. 48 h after transfection, cells were fixed in 4% PFA with 4% sucrose and processed for immunostaining as described above.

##### Quantification and Statistical Analysis

The immunofluorescence intensities of plasma membrane or cytoplasmic staining were measured using ImageJ. The intensity ratios were calculated and analyzed using GraphPad Prism 6. Student's *t* tests or one-way ANOVA^2^ with Tukey's post hoc tests were performed for hypothesis testing.

## RESULTS

### 

#### 

##### Ankyrin-B Is Excluded from the Lateral Plasma Membrane in Human Bronchial Epithelial Cells

Cultured epithelial cells require ankyrin-G for biogenesis of their lateral membranes (22). Ankyrin-G, in turn, requires interaction with βII-spectrin as well as palmitoylation for its function in lateral membrane biosynthesis (4, 23). Ankyrin-B recruits β-2 spectrin to an intracellular compartment in neonatal cardiomyocytes (7), but ankyrin-B has not been studied in epithelial cells. Therefore, we used human bronchial epithelial (HBE) cells to compare the localization of ankyrin-G and ankyrin-B.

**FIGURE 1.**
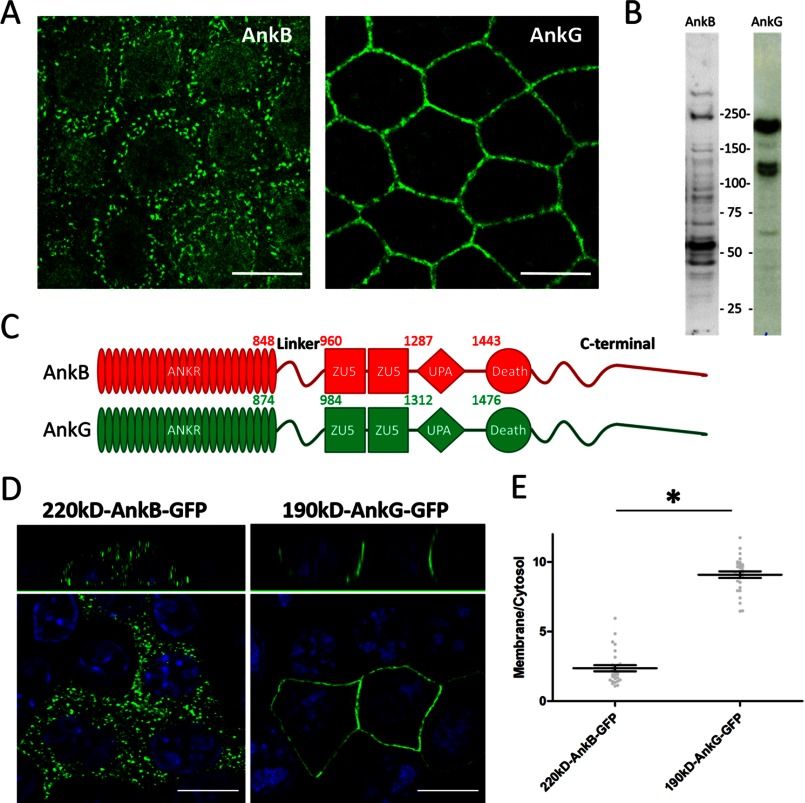
**AnkB is excluded from the lateral membrane of polarized HBE cells.** *A*, immunofluorescence showing total endogenous AnkB and AnkG in polarized HBE cells. *Scale bar* = 10 μm. *B*, immunoblot analysis of total HBE cell lysate showing various AnkB and AnkG isoforms. *C*, schematic representation of the canonical 220-kDa AnkB (*red*) and 190-kDa AnkG (*green*) polypeptides. *D*, immunofluorescence showing transiently expressed, GFP-tagged AnkB and AnkG (*green*, GFP staining; *blue*, DAPI staining) in polarized HBE cells. *Scale bar* = 10 μm. *E*, quantification of the immunofluorescence intensity ratio of plasma membrane *versus* cytosolic GFP staining (Student's *t* test, *n* = 23–25). *, *p* < 0.05.

We first determined the expression and localization of endogenous ankyrin-B and ankyrin-G in HBE cells. An antibody against the C-terminal domain of ankyrin-B detected multiple spliced isoforms, including the 220-kDa isoform and a major 55-kDa isoform (Fig. 1*B*). Immunofluorescence showed no ankyrin-B immunoreactivity on the plasma membrane and, instead, revealed intracellular vesicles of unknown identity (Fig. 1*A*). Three major ankyrin-G isoforms (one at 210 kDa and two at 120 kDa) are expressed in HBE cells (Fig. 1*B*) and are mainly concentrated on the plasma membrane, consistent with previous findings (*A*) (4, 22). We next compared the localization of transfected 220-kDa ankyrin-B-GFP and 190-kDa ankyrin-G-GFP in HBE cells. Fig. 1*C* is a schematic showing the structural details of ankyrin proteins, where ankyrin-B domains are highlighted in *red* and ankyrin-G domains are highlighted in *green*. 220-kDa ankyrin-B-GFP is excluded from the plasma membrane, whereas it is associated with vesicles in a similar pattern as endogenous ankyrin-B, and the 190-kDa ankyrin-G predominantly localizes to the lateral membrane (Fig. 1, *D* and *E*).

##### An ANK Repeat-ZU5 Linker Determines Lateral Membrane Exclusion of Ankyrin-B

**FIGURE 2.**
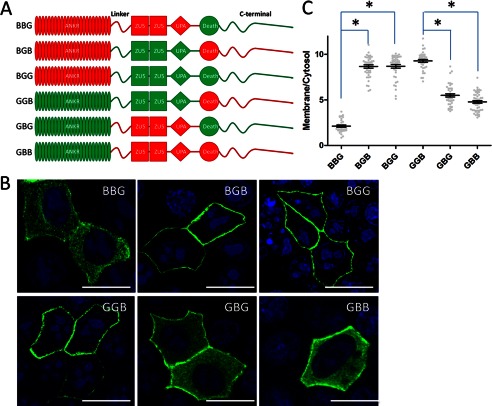
**The linker-ZU5_2_-UPA module of ankyrin proteins regulates their subcellular localization.** *A*, schematic representation of the AnkB/G chimeric proteins. *Red*, AnkB counterparts; *green*, AnkG counterparts. *B*, immunofluorescence showing transiently expressed, GFP-tagged AnkB/G chimeric proteins (*green*, GFP staining; *blue*, DAPI staining) in polarized HBE cells. *Scale bar* = 10 μm. *C*, quantification of the immunofluorescence intensity ratio of plasma membrane *versus* cytosolic GFP staining (one-way ANOVA followed by Tukey's post hoc test, *n* = 27–35). *, *p* < 0.05.

To explore the molecular mechanism for the dramatic difference between plasma membrane localization of ankyrin-B and ankyrin-G, we examined which domain is required for plasma membrane localization using B/G chimeras. We first used the previously generated ankyrin-B/G chimeric proteins containing all eight combinations of membrane-binding, spectrin-binding, and C-terminal domains (11). Surprisingly, the C-terminal region, which was reported to regulate the activity of ankyrin-B in neonatal cardiomyocytes (11), is not required for plasma membrane localization of ankyrin-B or ankyrin-G (, Fig. 2*A–C*, *BBG* and *GGB*). Instead, we found that the central spectrin-binding domain determines the specificity of ankyrin localization. Ankyrin-B, containing the ankyrin-G spectrin-binding domain (Fig. 2, *A–C*, *BGB*), efficiently targets the lateral membrane. Similarly, ankyrin-G, containing the ankyrin-B spectrin-binding domain, shows more intracellular localization (Fig. 2, *A–C*, *GBG*), even though it did not become completely intracellular.

**FIGURE 3.**
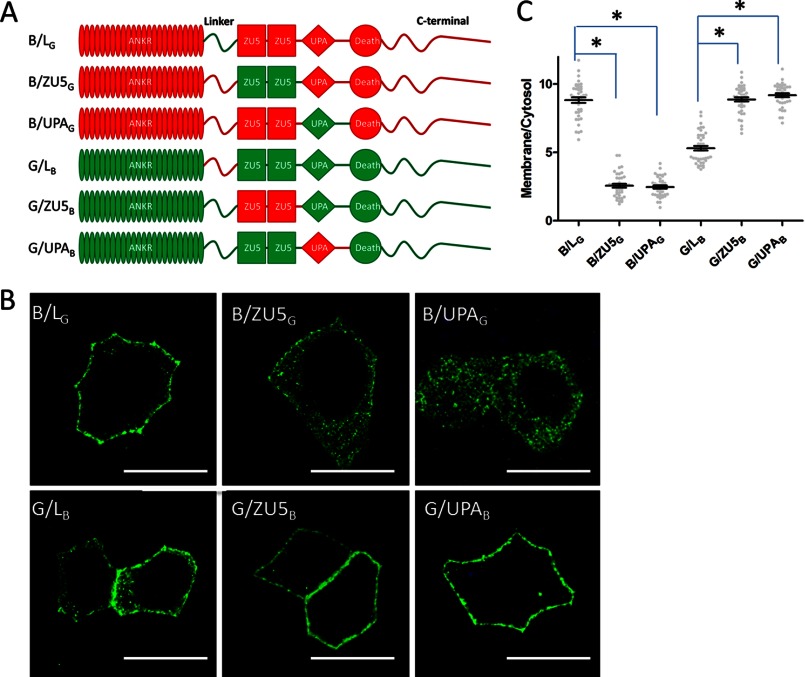
**An ANK repeat-ZU5 linker contains the information regulating AnkB or AnkG subcellular localization.** *A*, schematic representation of the second-generation AnkB/G chimeric proteins. *Red*, AnkB counterparts; *green*, AnkG counterparts. *B*, immunofluorescence showing transiently expressed, GFP-tagged second-generation AnkB/G chimeric proteins in polarized HBE cells. *Scale bar* = 10 μm. *C*, quantification of the immunofluorescence intensity ratio of plasma membrane *versus* cytosolic GFP staining (one-way ANOVA followed by Tukey's post hoc test, *n* = 30–37). *, *p* < 0.05.

The spectrin-binding domain consists of a linker peptide, two ZU5 domains, and a UPA domain. The β spectrin-binding site has been mapped to the first ZU5 domain (23, 25). To further explore the structural requirement for ankyrin localization in HBE cells, we made a second generation of ankyrin-B/G chimeric constructs in which we sequentially exchanged their linker peptides, ZU5 domains, and UPA domains. The sequence boundaries of each region were determined by the structure of the ZU5_2_-UPA superdomain module of ankyrin-B (6). We then compared the cellular localization of chimeras transfected into HBE cells. Interestingly, neither the ZU5 domains nor the UPA domain were required for localization of ankyrin-B or ankyrin-G because the chimeric proteins (B/ZU5_G_, B/UPA_G_, G/ZU5_B_, and G/UPA_B_) behaved the same as their wild-type counterparts (Fig. 3, *A–C*). However, exchange of the linker peptides (hereby referred to as the B-linker and G-linker for ankyrin-B and ankyrin-G, respectively) between ankyrin-B and ankyrin-G resulted in dramatic alteration of cellular localization. Ankyrin-B, containing the G-linker (*B/L_G_*), gained lateral membrane localization, whereas ankyrin-G containing the B-linker (*G/L_B_*) showed increased intracellular staining (Fig. 3, *A–C*).

##### The B-linker Acts through an Autoinhibitory Mechanism

**FIGURE 4.**
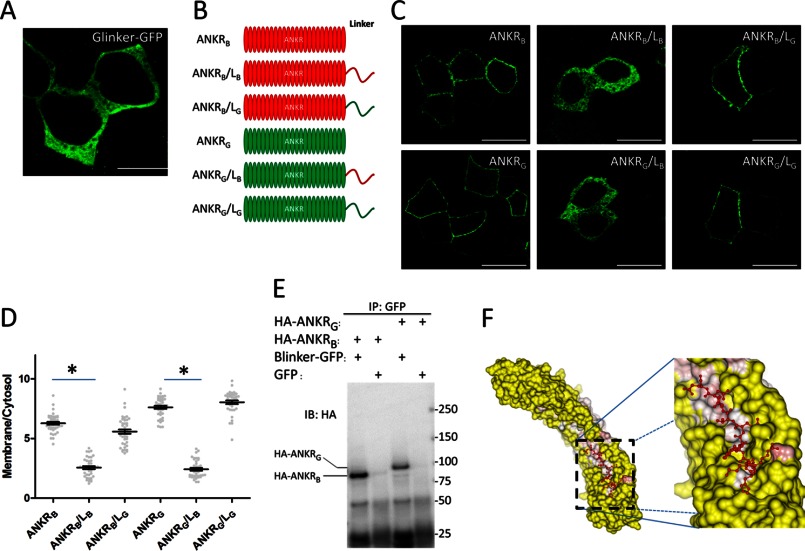
**The ANK repeat-ZU5 linker regulates AnkB localization through autoinhibition.** *A*, the linker region of AnkG (G-linker) was tagged with GFP and transiently expressed in HBE cells. Cells were fixed and stained against GFP to visualize G-linker localization. *Scale bar* = 10 μm. *B*, schematic representation of the truncated AnkB/G chimeric proteins. *Red*, AnkB counterparts; *green*, AnkG counterparts. *C*, immunofluorescence showing transiently expressed, GFP-tagged truncated AnkB/G chimeric proteins. *Scale bar* = 10 μm. *D*, quantification of the immunofluorescence intensity ratio of plasma membrane *versus* cytosolic GFP staining (one-way ANOVA followed by Tukey's post hoc test, *n* = 33–36). *, *p* < 0.05. *E*, coimmunoprecipitation (*IP*) of the B-linker and the ANK repeat domain. The HA-tagged ANK repeat domain of AnkB (*HA-ANKR_B_*) or AnkG (*HA-ANKR_G_*) was coexpressed with GFP-tagged B-linker (or GFP only as a negative control) in 293T cells. Samples were then immunoprecipitated with anti-GFP antibody and blotted (*IB*) with anti-HA antibody. *F*, structure of the human AnkR C-terminal 12 ankyrin repeats with a following peptide. The image was generated using CCP4MG software. The surface of the ankyrin repeats was colored by temperature factor, and the peptide was colored *red*.

Because ankyrin-B targets the plasma membrane when its linker region is replaced with the G-linker, we initially hypothesized that the G-linker itself associates independently with the plasma membrane. To test this hypothesis, GFP-tagged G-linker was expressed in HBE cells. However, the G-linker alone showed no membrane localization (Fig. 4*A*). We next generated truncated constructs only including the ANK repeat domain and the linker region (Fig. 4*B*). Surprisingly, the ANK repeat domain of either ankyrin-B or ankyrin-G was sufficient for lateral membrane targeting (ANKR_B_, ANKR_G_), and this membrane localization was not affected by addition of the G-linker (ANKR_B_/L_G_, ANKR_G_/L_G_) (Fig. 4, *C* and *D*). However, fusion of the B-linker to the C terminus of the ANK repeat domain efficiently blocked the lateral membrane binding activity of the ANK repeat domains of both ankyrin-B and ankyrin-G (ANKR_B_/L_B_, ANKR_G_/L_B_; Fig. 4, *C* and *D*).

Given the fact that the B-linker inhibits lateral membrane targeting of the ANK repeat domain, the B-linker may interact directly with ANK repeats. To test this hypothesis, we performed a coimmunoprecipitation assay in which GFP-tagged B-linker was coexpressed with the HA-tagged ANK repeat domain of ankyrin-B or ankyrin-G. After immunoprecipitating the GFP-B-linker, we detected robust HA immunoreactivity, which indicated a strong interaction between the B-linker and the ANK repeat domain (Fig. 4*E*). Interestingly, an atomic structure of the C-terminal 12 ankyrin repeats of ankyrin-R, solved by Michaely and colleagues (26), showed that the ankyrin repeat solenoid contains an extended groove that associates with the endogenous C-terminal peptide corresponding to the B-linker (Fig. 4*F*). This model suggests an autoinhibitory mechanism where an unstructured B-linker polypeptide represses the activity of ANK repeat domain through association with the ANK repeat groove, thus competing with membrane partners.

##### The B-linker Prevents Interaction of Ankyrin-B with E-cadherin and Neurofascin 186

**FIGURE 5.**
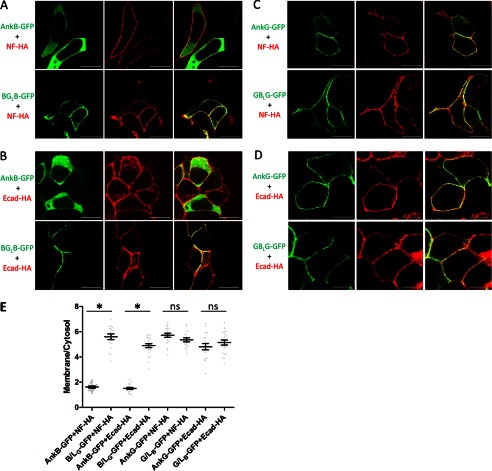
**The B-linker inhibits the interaction between AnkB and E-cadherin and neurofascin 186.** AnkB-GFP or B/L_G_-GFP (*A* and *B*) or AnkG-GFP or G/L_B_ -GFP (*C* and *D*) were coexpressed with HA-tagged neurofascin 186 (*NF-HA*) or E-cadherin (*Ecad-HA*) in HEK293 cells. Cells were then fixed and costained with anti-GFP (*green*) and anti-HA (*red*). *Scale bars* = 10 μm. *E*, quantification of the immunofluorescence intensity ratio of plasma membrane *versus* cytosolic GFP staining (one-way ANOVA followed by Tukey's post hoc test, *n* = 17–24). *, *p* < 0.05. *ns*, not significant.

Ankyrin-G is the native partner for neurofascin 186 and binds to E-cadherin with a much higher affinity than ankyrin-B (27–29). Therefore, we were interested in testing whether the B-linker functions by regulating the interaction of ankyrin-B with E-cadherin or neurofascin 186. We addressed this question using a previously reported cellular recruitment assay where coexpression of ankyrin-G with a membrane partner in HEK 293 cells results in recruitment of ankyrin-G from the cytoplasm to the plasma membrane (20). Ankyrin-B, when coexpressed with neurofascin 186 or E-cadherin, which both target to the plasma membrane in HEK293 cells, remained in the cytoplasm, indicating no interaction between ankyrin-B and neurofascin 186 or E-cadherin (Fig. 5, *A* and *B*). However, the chimeric ankyrin-B with the G-linker (*B/L_G_*) was efficiently recruited to the plasma membrane by both neurofascin 186 and E-cadherin (Fig. 5, *A* and *B*). These results imply that the B-linker suppresses the binding activity of ankyrin-B with neurofascin 186 and E-cadherin, probably through autoinhibition, as noted above.

In contrast to its effects on ankyrin-B, the B-linker, when substituted for the G-linker, did not completely block the recruitment of ankyrin-G by neurofascin 186 or E-cadherin (Fig. 5, *C* and *D*). This result is consistent with the cellular localization results where the B-linker only partially prevents ankyrin-G from targeting the lateral membrane in HBE cells (Fig. 3, *B* and *C*). One explanation for the different sensitivities of ankyrin-B and ankyrin-G may be that the B-linker requires specific regions in the ANK repeat domain of ankyrin-B for an efficient intramolecular interaction and repression. Consistent with this interpretation, the coimmunoprecipitation assay shows greater binding between the B-linker and the ANK repeat domain of ankyrin-B than that of ankyrin-G (Fig. 4*E*).

##### Multiple Sites in the B-linker Are Required for Its Inhibitory Function

To identify critical regions required for the autoinhibition activity of the B-linker, we first truncated the C-terminal half (residues 891–960) of the B-linker and found that it still maintained its inhibitory activity (data not shown). Then we performed NAAIRS scanning mutagenesis on the N-terminal portion (residue 848–890) of the B-linker (30). The NAAIRS (Asp-Ala-Ala-Ile-Arg-Ser) hexapeptide was identified in diverse secondary structures (31). This peptide, thus, is able to adopt a flexible conformation on the basis of the local protein structural context and has been widely used for efficient scanning mutagenesis by replacing six amino acids at a time without perturbing overall protein folding (32–36).

**FIGURE 6.**
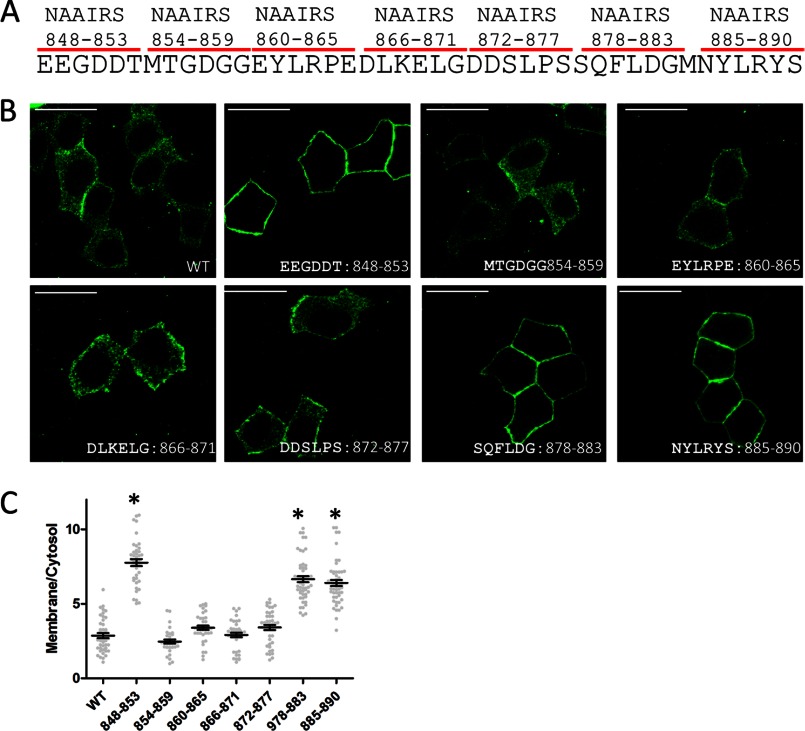
**Multiple sites are required for the inhibitory activity of the B-linker.** *A*, every six amino acids were mutated into NAAIRS to scan through the B-linker region. *B*, immunofluorescence showing transiently expressed, GFP-tagged AnkB mutants. *Scale bar* = 10 μm. *C*, quantification of the immunofluorescence intensity ratio of plasma membrane *versus* cytosolic GFP staining (one-way ANOVA followed by Tukey's post hoc test, *n* = 32–39). *, *p* < 0.05.

We identified three regions (amino acids 848–853, 878–883, and 885–890) within the B-linker that are required for lateral membrane exclusion of ankyrin-B. When those amino acids were mutated into NAAIRS, transiently expressed ankyrin-B-GFP predominantly localized on the lateral membrane in polarized HBE cells (Fig. 6). Considering our coimmunoprecipitation result showing that the B-linker interacts with the ANK repeat domain (Fig. 4*E*), this finding implies that the B-linker requires multiple contact sites for full interaction and repression of interactions.

##### The B-linker Determines the Plasma Membrane Localization of Ankyrin-B in Cultured Hippocampal Neurons

**FIGURE 7.**
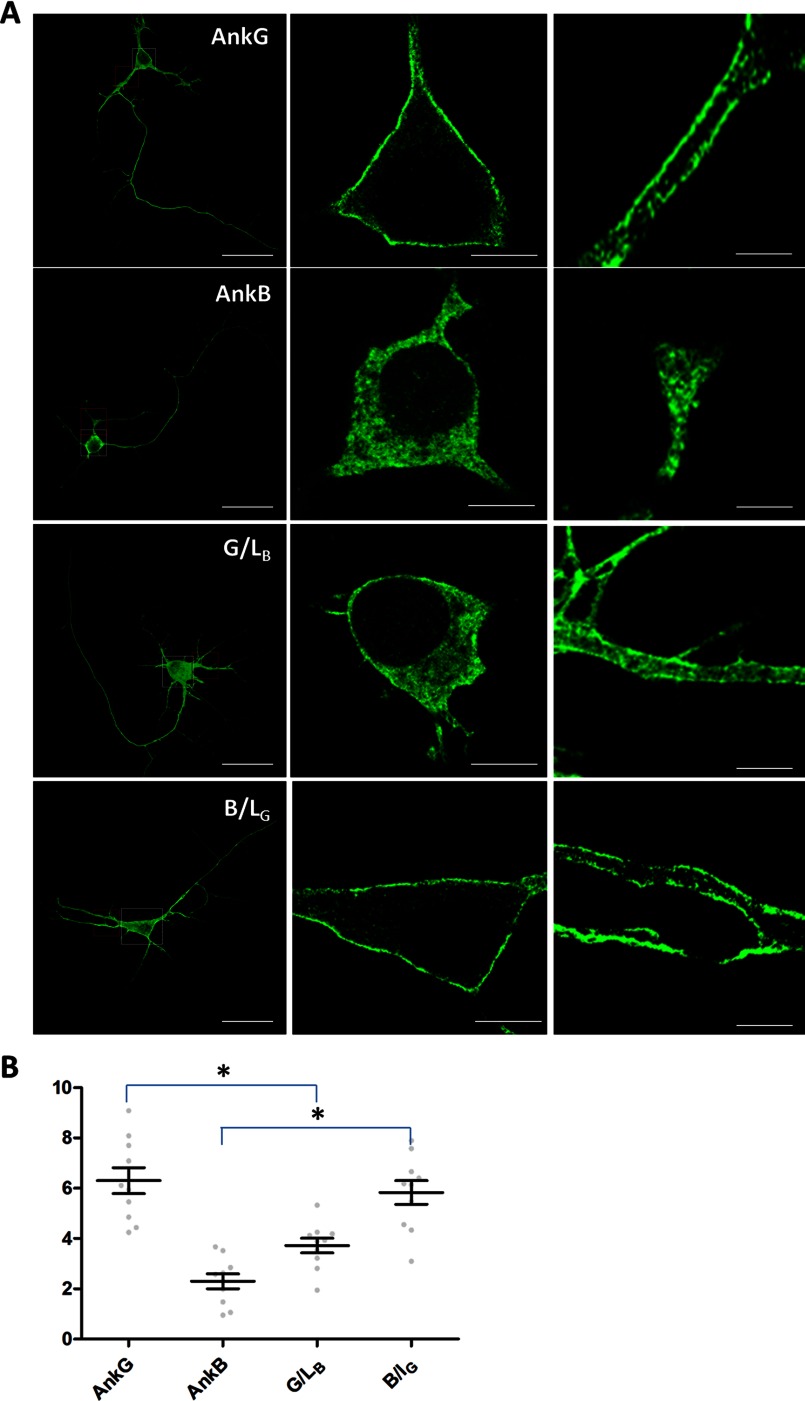
**The B-linker regulates ankyrin protein localization in the same way in neurons as in HBE cells.** *A*, immunofluorescence showing transiently expressed, GFP-tagged, second-generation AnkB/G chimeric proteins (see Fig. 4) in hippocampal neurons. The first column shows the profile of entire transfected neurons. *Scale bar* = 100 μm. The cell body (shown in the second column, *scale bar* = 5 μm) is boxed in *white*, whereas the primary dendrite (shown in the third column, *scale bar* = 2 μm) is boxed in *red. B*, quantification of the immunofluorescence intensity ratio of plasma membrane *versus* cytosolic GFP staining (one-way ANOVA followed by Tukey's post hoc test, *n* = 10). *, *p* < 0.05.

We next investigated whether the B-linker autoinhibition mechanism extends to neurons. We transfected wild-type, 220-kDa ankyrin-B-GFP, 190-kDa ankyrin-G-GFP, and their linker chimeras into cultured hippocampal neurons and compared their membrane localization in soma and primary dendrites. We were unable to resolve the plasma membrane from cytoplasm in axons because of their small size. However, consistent with results in HBE cells, we found that 190-kDa ankyrin-G-GFP was able to target to the plasma membrane of soma and primary dendrites (Fig. 7*A*, *AnkG*, and *B*), whereas 220-kDa ankyrin-B predominantly localized in the cytoplasm in these cells (*A*, *AnkB*, and *B*). However, ankyrin-G with the B-linker showed increased cytoplasmic staining (Fig. 7*A*, *G/L_B_*, and *B*), whereas ankyrin-B with the G-linker, conversely, exhibited robust membrane staining (*A*, *B/L_G_*, and *B*).

##### The Active Region of the B-linker Evolved from a Single, Highly Divergent Exon

**FIGURE 8.**
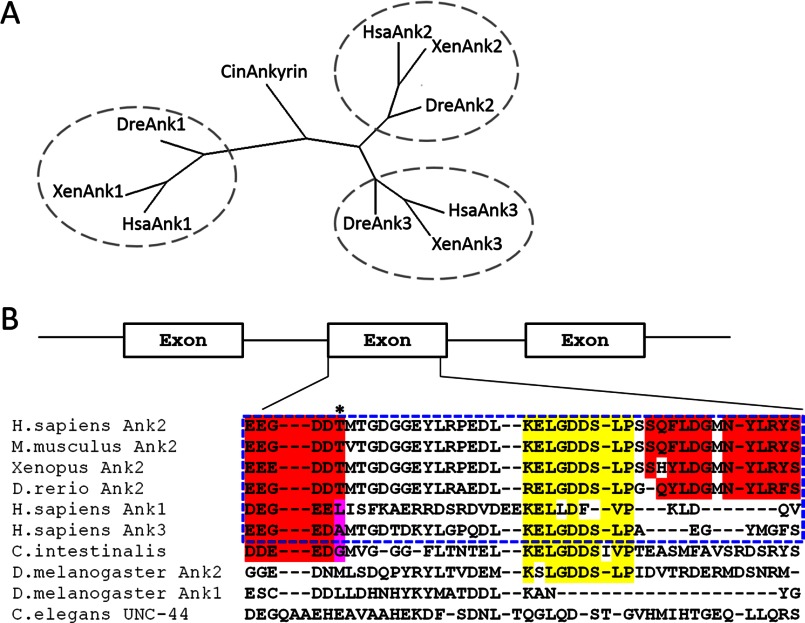
**The B-linker region is encoded by a single exon evolutionarily conserved in the ankyrin-B lineage.** *A*, a phylogenetic tree showing the evolutionary lineage of ankyrin proteins. *B*, sequence alignment of the linker region in AnkB across multiple species and other ankyrin isoforms. Regions highlighted in *red* represent the critical regions for the inhibitory activity of the linker, and regions highlighted in *yellow* represent conservation back to ancient ankyrin molecules in *Drosophila*.

The similar role of the B-linker in regulating plasma membrane localization in both HBE cells and hippocampal neurons suggests that the autoinhibition mechanism may contribute to functional differences between ankyrin family members. The three vertebrate ankyrins evolved from a single ankyrin gene present in the urochordate *C. intestinalis* ((2), Fig. 8*A*). Surprisingly, the critical region of the B-linker (Fig. 6*A*) required for restricting membrane localization is encoded by a single exon in all three vertebrate ankyrins (Fig. 8*B*). The B-linker exon is highly conserved within the Ank2 family across multiple species, from humans to fish (*Homo sapiens*, *Mus musculus*, *Xenopus*, and *Danio rerio*, Fig. 8*B*), especially in the regions identified to be critical for its inhibitory function (*B*, highlighted in *red*). On the other hand, the linker exons are divergent among Ank1, Ank2, and Ank3 (*H. sapiens* Ank1, Ank2, and Ank3, Fig. 8*B*), although conserved core elements indicate a common evolutionary origin (*B*, highlighted in *yellow*).

## DISCUSSION

Ankyrin-B is primarily associated with an intracellular compartment(s), whereas ankyrin-G localizes to plasma membranes in epithelial cells (Fig. 1) as well as cardiomyocytes (7, 37). Here we identify an ankyrin-B-specific linker peptide connecting the ANK repeat domain to the ZU5_2_-UPA module that inhibits binding of ankyrin-B to membrane protein partners E-cadherin and neurofascin 186 and prevents association of ankyrin-B with epithelial lateral membranes as well as neuronal plasma membranes. The active region of the B-linker is encoded by a small exon that is highly divergent between different ankyrin family members but conserved in the ankyrin-B lineage. We show that the ankyrin-B linker can suppress activity of the ANK repeat domain through an intramolecular interaction, likely with a groove on the surface of the ANK repeat solenoid, thereby regulating the affinities between ankyrin-B and its binding partners. Scanning mutagenesis revealed three regions in the ankyrin-B linker that are required for its full autoinhibition. These results provide a simple evolutionary explanation for how ankyrin-B and ankyrin-G have acquired striking differences in their plasma membrane association while maintaining overall high levels of sequence similarity.

Deficiency of ankyrin-B causes a constellation of traits termed “ankyrin-B syndrome” (38–41). Previous research has identified multiple human genetic ankyrin-B variants (E1425G, L1622I, T1626N, R1788W, and E1813K) (40–43) that are associated with this syndrome. Interestingly, a search of the Exome Variant Server revealed a human genetic variant in this linker region of ankyrin-B (NM_020977.3). This variant causes a glutamine (amino acid 879)-to-arginine mutation that happens to be located in one of the three critical regions of B-linker that we identified through scanning mutagenesis (Fig. 6). Therefore, it will be interesting to test whether this mutation causes any cellular or physiological defects in future experiments. Additionally, a study conducted by Cunha and colleagues (44) revealed multiple new exons in the ANK2 gene, and one of those exons is immediately 5′ to the B-linker exon. This new exon encodes a short peptide that can potentially alter the relative position between the ANK repeat domain and the B-linker. Similar alternative exons were also identified 5′ to the G-linker exon (45), which may affect membrane protein interactions of ankyrin-G polypeptide.

Here we show that endogenous ankyrin-G localizes to the plasma membrane, whereas ankyrin-B exists in some intracellular vesicles with unknown identity in HBE cells. However, membrane association may be cell type-specific because in some cases both ankyrin-B and ankyrin-G localize to the plasma membrane, although in different subdomains. For example, in myelinated neurons, ankyrin-G targets the nodes of Ranvier, whereas ankyrin-B localizes to the paranode (19). Another example is the cardiac T-tubules membrane, where ankyrin-B localizes together with Na/K ATPase, the Na/Ca exchanger, and the InsP_3_ receptor (46), whereas ankyrin-G localizes to both the intercalated disc and T-tubules and is required for targeting of Na_v_1.5channels (47). These specific localization patterns may be achieved through ankyrin-B and ankyrin-G interactions unaffected by the B-linker peptide and likely involve additional proteins.

Ankyrins recognize their membrane partners through natively unstructured peptides in their cytoplasmic domains (3). A structure of the C-terminal half of the ankyrin-R ANK repeat domain revealed a groove-like surface that provides a potential binding site for unstructured peptides (26). The linker we identified here may function as a native competitor or regulator for determining binding affinities with different partners. To further confirm this hypothesis, it will be important in the future to determine the atomic structures of the ANK repeats of ankyrin-B with membrane partners and with the B-linker.

We showed previously that ankyrin-G is *S*-palmitoylated at cysteine 70 in the ANK repeat domain (4), which is required for plasma membrane localization in unpolarized Madin-Darby canine kidney cells. Interestingly, here we found that fusion of the B-linker to the ANK repeat domain of ankyrin-G or ankyrin-B reduces their lateral membrane targeting. One explanation may be that the B-linker interferes with *S*-palmitoylation because protein *S*-palmitoylation is mediated by a group of palmitoyltransferases that can transiently associate with their substrates and transfer palmitic acids to target cysteine(s) (48). Another possibility is that palmitoylation follows association of ANK repeats with membrane proteins. In this case, the B-linker prevents ANK repeat domains from binding to plasma membrane compartments where they are subsequently palmitoylated.

Ankyrins and their partner, spectrin, have from their initial discovery exhibited association with both plasma membranes and intracellular organelles (24, 49–51). Our discovery of a mechanism for preventing association of ankyrin-B with plasma membranes through intramolecular competition for membrane binding partners provides a molecular clue as to how closely related ankyrins can direct spectrin to both cytoplasmic compartments and the plasma membrane. Given that ankyrin-B and ankyrin-G originated from a single ancestral ankyrin, it is possible that prechordate ankyrin had both intracellular and plasma membrane-related functions. The identity of the ankyrin-B-associated intracellular structures in cardiomyocytes (7) or HBE cells (Fig. 1*A*) remains unknown. Therefore, it will be intriguing to identify the composition and function of ankyrin-B-positive organelles and their cellular functions.

## Footnotes

2 The abbreviations used are:

ANOVA: analysis of variance
HBE: human bronchial epithelial
AnkB: ankyrin-B
AnkG: ankyrin-G
ANK: ankyrin.
